# The Water Cure in the Bedroom; or, Hydropathy at Home

**Published:** 1892-06

**Authors:** 


*The Water Cure in the Bedroom; or, Hydropathy at Home.* By G. H. DOUDNEY, M.B. Pp. 46. Bristol: JOHN WRIGHT & CO. [1891.]

Hydropathy, relieved of the taint of charlatanism and mystery, is becoming one of the most valuable aids to modern treatment, and although many of the complicated processes used in hydropathic treatment must of necessity be carried out in establishments fitted up for the purpose, much may be done in an ordinary bedroom with simply the apparatus usually contained therein. This book shows how much may be accomplished in the treatment of simple ailments which commonly do not require medical aid, and its object is a laudable one, that of substituting baths, fomentations, pads, etc., for the domestic drugging which is far too common.

